# Editorial: Exercise as a central pillar for targeted health and performance

**DOI:** 10.3389/fphys.2026.1867393

**Published:** 2026-05-19

**Authors:** Rodrigo Zacca, Justin Roberts, Ana Isabel Padrão, Liliana C. Baptista

**Affiliations:** 1 Research Center in Physical Activity, Health and Leisure (CIAFEL), Faculty of Sports, University of Porto (FADEUP), Porto, Portugal; 2 Laboratory for Integrative and Translational Research in Population Health (ITR), Porto, Portugal; 3 Laboratory of Sport Physiology, Faculty of Sports, University of Porto, Porto, Portugal; 4 Nucleus of Research in Human Motricity Sciences, Universidad Adventista de Chile, Chillán, Chile; 5 Cambridge Centre for Sport and Exercise Sciences, Faculty of Science and Engineering, Anglia Ruskin University, Cambridge, United Kingdom; 6 Interdisciplinary Center for the Study of Human Performance (CIPER-UC), Faculty of Sports Sciences and Physical Education (FCDEF), University of Coimbra, Coimbra, Portugal

**Keywords:** exercise training, health promotion, integrative physiology, sport performance, targeted exercise

Exercise is an essential component in supporting and optimizing health, wellbeing and performance (Qiu et al., 2023). Based on an integrative systems biology approach, regular exercise training provides progressive and meaningful adaptations to our cardiovascular, respiratory, gastrointestinal, musculoskeletal, immune and nervous systems to name a few (Silverman and Deuster, 2014). In doing so, the type of exercise undertaken can positively impact health from metabolism to mood, from cognitive function to functional wellbeing. Modulating the intensity, duration, volume, frequency and even modality of exercise can have significant benefits in terms of optimizing body composition (via lean to fat mass ratios), supporting muscle protein synthesis and bone density, through to metabolic flexibility and immunological resilience.

The major non-communicable diseases (NCDs) which can impact health and wellbeing include: obesity, type 2 diabetes, cancer and cardiovascular diseases (Freihat et al., 2025). Additionally, neurological and mental health disorders (including depression), as well as musculoskeletal diseases (including osteoarthritis, chronic back pain) are also classed as NCDs which impact health and quality of life with age. Physical exercise when performed regularly as part of a lifestyle strategy can literally act “as medicine” (Pedersen and Saltin, 2015) both therapeutically and preventatively through improvements in blood glucose and lipid profiles, insulin sensitivity and regulation, prostaglandin and antioxidant mediation driving positive adaptations at a cellular, tissue and system level. As such, exercise can have subsequent impact on reducing the need for on-going medication use. Resistance training is widely regarded as a central component to impact anabolic signaling during the post-exercise recovery phase, supporting muscle adaptations which may also help to minimize the deleterious effects of sarcopenia observed with aging. Likewise, cardiovascular exercise can positively impact cardiorespiratory efficiency and capacity, supporting functional health, movement economy and quality of life.

From a performance perspective, exercise when undertaken in accordance with contemporary exercise physiology approaches (including progressive overload, training specificity and adequate recovery) can significantly impact neuromuscular and physiological adaptations driving “supercompensatory” approaches leading to performance gains. Whether the individual targets strength, power, speed or endurance, the training modality, type, frequency, volume and intensity of exercise can impact the nature of overload or conversely underperformance. Recent interest in high-intensity interval training and cross-fit exercise has furthered interest in targeted use of training specificity to benefit individuals across the lifespan who likely have differing health or performance goals.

This Research Topic therefore aimed to investigate how different exercise modalities and approaches can serve as a foundational intervention in managing chronic diseases through to enhancing athletic performance. This Research Topic features 14 articles, mostly from China, including 7 systematic reviews, 6 original research papers and 1 narrative review. In this editorial, we provide a summary of the key findings from each manuscript.

## Exercise supporting obesity and pre-diabetes

There is current concern with the increasing prevalence of obesity and diabetes, particularly within children and adolescent populations. Both conditions place considerable strain on healthcare systems, further impacting cardiovascular and metabolic health, and longer-term risks associated with declining health. Minimizing or offsetting prevalence of obesity and/or diabetes is therefore an important consideration for longer-term healthcare. However, with additional concerns that many younger individuals fail to meet physical activity guidelines, strategies to encourage shorter duration/high intensity bouts of exercise may be one approach to proactively engage younger people in sustaining healthy lifestyles. Ye et al. compared low-volume high-intensity interval training (LV-HITT) versus moderate-intensity continuous training (MICT) or no exercise on cardiometabolic health (including body composition) in overweight and obese children and adolescents. From this systematic review of 12 trials (with 609 collated participants), LV-HITT was effective in improving body composition, systolic blood pressure and maximal exercise capacity more so than either no exercise or MICT, although both MICT and LV-HIIT were comparable in terms of effective body fat reduction. Similar findings were also reported by Zheng et al. with recommendations that running-based HIIT three times a week can be an effective strategy, although cycling-based exercise offers a safer approach. Such findings have implications for time effective exercise strategies in overweight and obese children and adolescents.

In contrast, use of MICT would appear to offer superior benefits when considering adult populations with pre-diabetes. In a randomized controlled trial, Chen et al. assessed MICT or HIIT strategies performed 3x per week over 12 weeks in 71 adults with pre-diabetes. MICT significantly impacted visceral adipose and body fat compared with HIIT, along with positive effects on diastolic blood pressure and triglyceride levels. Interestingly, both strategies significantly decreased fasting blood glucose and 2h glucose concentrations within group, but only HIIT had a within group increase in HbA1c. These findings pinpoint the use of MICT as a targeted approach to maximize health benefits of exercise in adult pre-diabetic populations.

## Exercise adherence and management of chronic back pain

Lower back pain is recognized as one of the most common NCDs and a leading cause of disability (Owen et al., 2020). In the UK alone, it has been estimated that direct and indirect costs associated with lower back pain are £12 billion per annum (Dagenais et al., 2008) adding to overall healthcare burden. There also appears to be a worrying upward trend in chronic low back pain in children and adolescents (Balagué et al., 1999), with evidence of recurrence or chronic pain being experienced. With negative implications for neurodevelopmental processes, educational absenteeism, physical activity avoidance and social behavioral problems, an assessment of the potential benefits of exercise therapy is warranted. Therefore, Jiang et al. undertook a systematic review and meta-analysis of randomized controlled trials (RCTs) to determine the effectiveness of exercise therapy alone or when combined with other treatments in reducing non-specific chronic low back pain in children and adolescents (aged 6–19 years). Despite limited evidence and substantial heterogeneity, the effect of exercise-based interventions alone demonstrated an uncertain effect on pain intensity. Meanwhile, combing exercise with other adjunct therapies such as spinal manipulative therapy, vibratory stimulation, or individualized physical therapy were associated with a small effect on pain intensity. This highlights the need for wider research aligned with each of these therapies particularly in younger age groups.

In a similar manner, Tan et al. investigated the effects of exercise therapy as well as adherence to the American College of Sports Medicine (ACSM) guidelines on treatment outcomes in adult patients with lower back pain. Based on a systematic review and meta-analysis of 36 studies (with 2284 collated participants), the authors found that exercise therapy had a significant beneficial effect on pain and disability reduction, with a greater effect if participants had a higher level of adherence to ACSM recommendations (including frequency, intensity and duration, and considering cardiorespiratory, resistance and flexibility exercises). This study highlights the importance of targeting exercise strategies which are more aligned with current recommendations for effective therapeutic benefits. Such findings are further supported from a systematic review and meta-analysis by Li et al. across 30 studies (with 2105 collated participants) also highlighting the therapeutic benefits of exercise on relieving pain, improving spinal dysfunction, including scoliosis correction. The authors found that the Zongjianji 18-poses (stimulating spinal muscles and ligaments involved in posture and movement) and Baduanjin approaches (a classical form of Qigong comprising 8 flowing exercises) offer higher effect sizes, with duration (10-30mins) and frequency (3-4x/week for 10–20 weeks) offering targeted benefits for spinal health.

## Novel exercise approaches for health promotion

With interest in how exercise can modulate immune function, little is known around the benefits of physical activity involving swinging patterns including sports/activities such as table tennis, badminton or volleyball. Such activities involve short bursts of high-intensity efforts, as well as variable motor skills and rapid decision-making. Moving away from the traditional effects of aerobic/anaerobic effects, Guodong et al. evaluated 14 studies (with 440 collated participants) using an integrated meta-analysis and machine learning approach to explore effects of swinging exercises on immune biomarkers. Findings indicated that swinging exercises (irrespective of age) can induce a dual immune response involving T-cell suppression and enhanced humoral immunity. This has implications for personalizing activities based on the author’s recommendations for integrated immune profiling, especially considering responders were characterized by a balanced immune profile.

For those who struggle with time or physical constraints, Ban et al. explored the potential benefits of zero-time exercise (ZETx) whereby participants can undertake a series of brief low to moderate intensity physical exercises which can be easily undertaken within normal daily routines (e.g. stretching, walking, skipping, stair climbing). This effectively forms the concept of “exercise snacking” built into daily activities with accumulated benefits. This narrative review proposes that ZETx can have multiple physiological benefits ranging from improved cardiorespiratory and metabolic function, to cognitive and emotional wellbeing. This has implications for developing a future blueprint involving standardized guidelines in employing ZETx strategies for those with chronic diseases or other constraints.

From a different angle, Lei et al. investigated the cardiometabolic health benefits of engagement in recreational football in 8–17 year olds (over 20 studies, with 2906 collated participants). Not only does participation in recreational team activities promote social interaction and sustained exercise adherence but actively uses variable aerobic and anaerobic energy systems. Findings demonstrated significant beneficial effects on body composition, body fat percentage, systolic blood pressure and triglycerides, but not cardiorespiratory fitness (e.g., peak oxygen uptake; $\dot{V}$O_2peak_). Whilst quality of evidence was considered low, the authors indicated that active engagement in recreational team activities such as football offers health-promoting benefits, particularly lending to the development of longer term (12-week), higher frequency (2–3 sessions per week) strategies. For younger individuals, Alotaibi et al. also note that use of a handgrip strength test could serve as a simple approach to monitor musculoskeletal fitness (significantly correlating with physical activity time, and performance measures such as standing long jump and 20m shuttle run test) as demonstrated in 118 male children aged 10–13 years. Such practical strategies could be easily employed in health monitoring programs or by coaches to evaluate developmental, health or performance progress for younger individuals.

## Optimizing sports training through different loading approaches

Whilst plyometric training is an effective and vital component to enhancing jump performance, Li et al. investigated whether increasing frequency of plyometric training was beneficial or not in male high and long jump athletes (n=39). Performance measured by isometric mid-thigh pull, countermovement jump, squat jump and standing long jump were all improved across a 4-week training phase in those who underwent 2 plyometric sessions per week and tapered effectively in the final week. For those undertaking 3 sessions per week, acute performance was negatively impacted prior to recovery in the taper week. This has implications for coaches/athletes in terms of regular monitoring of training load, muscle soreness and acute performance decrements when determining appropriate training loads for such athletes.

Zhou et al. also demonstrate that monitoring of physiological training and mechanical loads are important for assessing overall aerobic, strength and power attributes in sports involving complex strength and aerobic fitness patterns such as gymnastics. Integrating assessment of both external (e.g. jump volume) and internal (e.g. heart rate or perceived exertion) loads likely plays a more grounded approach to monitoring individual athlete responses to training phases.

Short sprint interval training (SSIT) is also a recognized method for enhancing aerobic and anaerobic capacity, but whether there is transference to explosive performance is less known particularly in sports such as basketball. Cao et al. therefore explored whether high intensity SSIT was more beneficial over 8 weeks than either moderate or multiple intensity SSIT in 36 female collegiate basketball players. Findings from this randomized controlled trial demonstrated benefits on explosive power, sprint performance and fatigue resistance when employing varied intensity SSIT formats as part of conditioning programs tailored to the demands of the sport. In a similar manner, Yue et al. compared findings from 20 studies (with 399 collated participants) to demonstrate that HIIT training can effectively improve aerobic capacity, peak and maximal power, thereby strategically benefiting Olympic combat sports based on the intermittent nature and demands of the sport. Both studies highlight the importance of using targeted exercise approaches to personalize training programs and maximize athlete gains specific to the demands of the respective sport.

**Figure 1:**
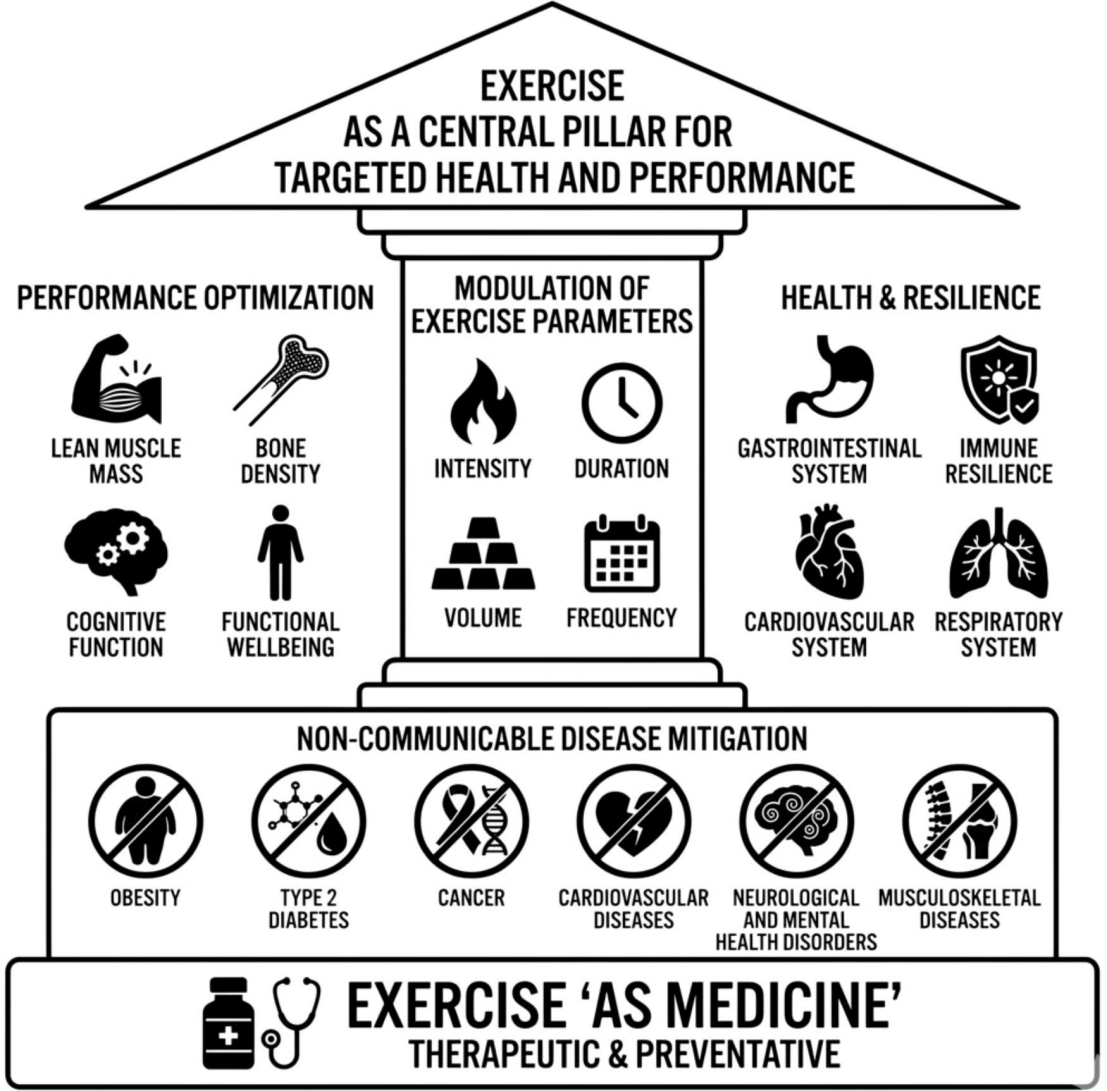
Overview highlighting how exercise acts as medicine by mitigating various non-communicable diseases, and its central role in enhancing parameters of health and physical performance.

In summary, the evidence presented in this Research Topic supports the notion that exercise should not be seen merely as a complementary practice, but as a fundamental multisystemic intervention, a “natural polypill” acting at the intersection of molecular biology and functional performance. From an integrative physiology perspective, it is well established that the precise manipulation of training variables, such as volume, intensity, and specificity, triggers adaptations ranging from antioxidant mediation and improved insulin sensitivity to the anabolic signaling required to mitigate sarcopenia, among other key adaptations. By underscoring the role of exercise in both the prevention and treatment of non-communicable diseases (NCDs) and the induction of physiological supercompensation, this Editorial reiterates that exercise, when prescribed based on contemporary (and classic) physiological principles, is the central pillar for optimizing health, immunological resilience, and human capacity. We hope the discussions initiated here will stimulate further research to refine “exercise as medicine” protocols, ensuring that it reaches its maximum potential in promoting health, sports performance and longevity (**Figure 1**).

## References

[B1] Balagué F. Troussier B. Salminen J. J. (1999). Non-specific low back pain in children and adolescents: risk factors. Eur. Spine Journal: Off. Publ. Eur. Spine Society Eur. Spinal Deformity Society Eur. Section Cervical Spine Res. Soc. 8, 429–438. doi:10.1007/s005860050201. PMID: 10664299 PMC3611213

[B2] Dagenais S. Caro J. Haldeman S. (2008). A systematic review of low back pain cost of illness studies in the United States and internationally. Spine Journal: Off. J. North. Am. Spine Soc. 8, 8–20. doi:10.1016/j.spinee.2007.10.005. PMID: 18164449

[B3] Freihat O. Sipos D. Aamir M. Kovacs A. (2025). Global burden and future projections of non-communicable diseases, (2000-2050): Progress toward SDG 3.4 and disparities across regions and risk factors. PloS One 20, e0336036. doi:10.1371/journal.pone.0336036. PMID: 41370213 PMC12694828

[B4] Owen P. J. Miller C. T. Mundell N. L. Verswijveren S. J. J. M. Tagliaferri S. D. Brisby H. . (2020). Which specific modes of exercise training are most effective for treating low back pain? Network meta-analysis. Br. J. Sports Med. 54, 1279–1287. doi:10.1136/bjsports-2019-100886. PMID: 31666220 PMC7588406

[B5] Pedersen B. K. Saltin B. (2015). Exercise as medicine - evidence for prescribing exercise as therapy in 26 different chronic diseases. Scandinavian J. Med. Sci. Sports 25, 1–72. doi:10.1111/sms.12581. PMID: 26606383

[B6] Qiu Y. Fernández-García B. Lehmann H. I. Li G. Kroemer G. López-Otín C. . (2023). Exercise sustains the hallmarks of health. J. Sport Health Sci. 12, 8–35. doi:10.1016/j.jshs.2022.10.003. PMID: 36374766 PMC9923435

[B7] Silverman M. N. Deuster P. A. (2014). Biological mechanisms underlying the role of physical fitness in health and resilience. Interface Focus 4, 20140040. doi:10.1098/rsfs.2014.0040. PMID: 25285199 PMC4142018

